# Contamination, Detection and Control of Mycotoxins in Fruits and Vegetables

**DOI:** 10.3390/toxins14050309

**Published:** 2022-04-27

**Authors:** Mina Nan, Huali Xue, Yang Bi

**Affiliations:** 1 College of Science, Gansu Agricultural University, Lanzhou 730070, China; nanmn@gsau.edu.cn; 2 Basic Experiment Teaching Center, Gansu Agricultural University, Lanzhou 730070, China; 3 College of Food Science and Engineering, Gansu Agricultural University, Lanzhou 730070, China

**Keywords:** patulin, Alternaria toxin, ochratoxin A, trichothecenes, adsorption, degradation

## Abstract

Mycotoxins are secondary metabolites produced by pathogenic fungi that colonize fruits and vegetables either during harvesting or during storage. Mycotoxin contamination in fruits and vegetables has been a major problem worldwide, which poses a serious threat to human and animal health through the food chain. This review systematically describes the major mycotoxigenic fungi and the produced mycotoxins in fruits and vegetables, analyzes recent mycotoxin detection technologies including chromatography coupled with detector (i.e., mass, ultraviolet, fluorescence, etc.) technology, electrochemical biosensors technology and immunological techniques, as well as summarizes the degradation and detoxification technologies of mycotoxins in fruits and vegetables, including physical, chemical and biological methods. The future prospect is also proposed to provide an overview and suggestions for future mycotoxin research directions.

## 1. Introduction

Fruits and vegetables are important nutritional sources for human and are one of the most important parts of the human diet. However, the production loss of fruits and vegetables caused by postharvest diseases was 35–55%, and in developing countries the loss even reached 55% [1]. Except for pesticide residues and heavy metals, the contamination of mycotoxins is another key reason that can cause a loss of production and a threat to the health and lives of consumers [2,3]. Mycotoxins are a secondary metabolite produced by filamentous fungi, which can naturally occur during growth, harvest, storage, transportation and processing [4]. About 150 fungi have the ability to produce more than 400 mycotoxins, such as trichothecenes (TCs), ochratoxin A (OTA), patulin (PAT), Alternaria toxins (ATs), etc. [5]. All these toxins have different chemical structures, but in common most of them have the toxic effects of DNA damage and cytotoxicity. At a low concentration, they can cause lesions in the liver, kidneys and gastrointestinal tract, and they even have carcinogenic, teratogenic and mutagenic effects [6,7]. The accumulation of mycotoxins in fruits and vegetables is not only a potential threat to human and animal health but also results in serious economic losses. Therefore, it is very important to minimize the potential risks to humans and economic losses through the monitoring, detection and control of mycotoxins.

In this review, we focus on the main mycotoxins (TCs, OTA, PAT and ATs) produced by major mycotoxigenic fungi (*Alternaria*, *Aspergillus*, *Penicillium* and *Fusarium*) in fruits and vegetables. The mycotoxin detection methods including chromatography coupled with a detector (i.e., mass, ultraviolet, fluorescence, etc.) technology and electrochemical biosensors technology are summarized, as well as the mycotoxin degradation and detoxification strategies in fruits and vegetables.

## 2. Occurrence, Contamination and Toxicity of Mycotoxins

**Figure 1:**
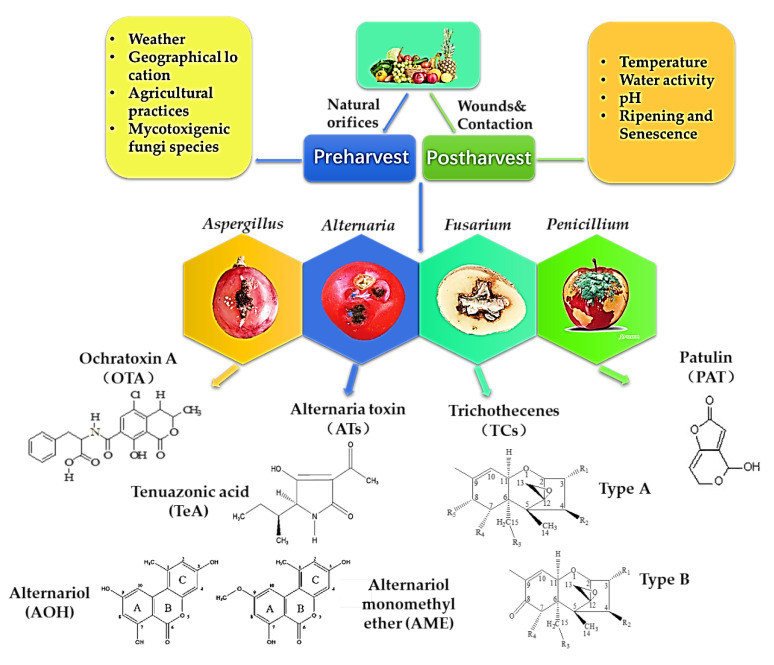
Mycotoxin structure and factors affecting fungi and toxin production in fruits and vegetables.

Different mycotoxins have been found not only in fruits and vegetables but also in their derivative products [8,9,10,11,12]. The mycotoxins commonly found in fruits and vegetables can be divided into four major categories: PAT, TCs, OTA and ATs. The toxicity, occurrence and contamination status of these mycotoxins are related to the fungi and hosts, as well as the environmental conditions [4]. The main route of infection of mycotoxigenic fungi to fruit and vegetable is that they can successfully colonize the host through wounds or natural orifices on the surface of fruits and vegetables before harvest. During the growth period, the host has strong resistance to exogenous, biological or abiotic stress. However, due to the large energy consumption at the postharvest stage, the resistance effect of fruits and vegetables is significantly weakened. Once the temperature and humidity conditions are appropriate, the pathogen can grow and colonize rapidly. Then, the secondary metabolite pathogenic mycotoxins are produced in the medium and late stages of growth [1]. The main factors related to the occurrence of fungi and toxin production in fruits and vegetables are shown in Figure 1.

### 2.1. Ochratoxin A (OTA)

**Figure 2:**
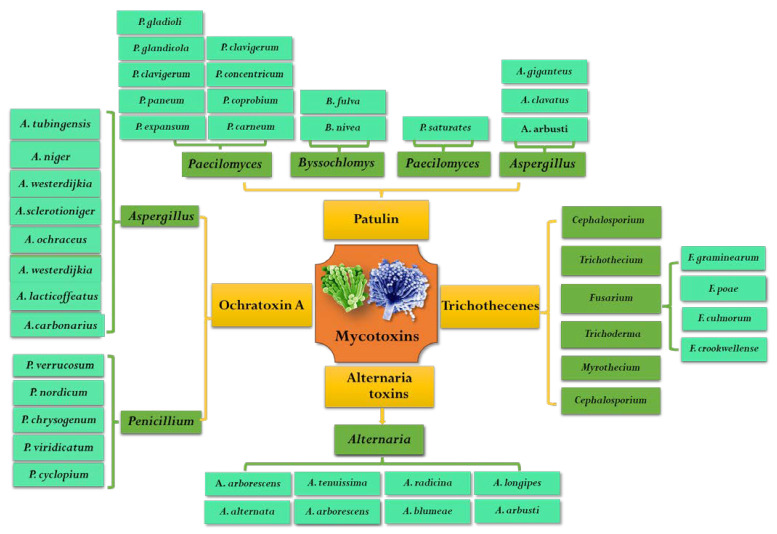
Common species of mycotoxigenic fungi in fruits and vegetables.

OTA was first isolated from *Aspergillus ochraceus* in South Africa in 1965 [13]. Since then, it was found that many species of *Aspergillus* and *Penicillium* fungi can produce OTA in different hosts. These fungi that are capable of producing OTA mainly include nine species of fungi belonging to two sections of *Aspergillus* and two species of *Penicillium*. OTA-producing species belonging to the *Circumdati* section were *A. ochraceus*, *A. westerdijkiae* and *A. steynii*. Moreover, six OTA-producing species in the *Aspergillus* section *Nigri* are *A. carbonarius*, *A. niger*, *A. lacticoffeatus*, *A. sclerotioniger* and *A. tubingensis* [14]. Because *Penicillium* species are more diverse in numbers and habitat range than *Aspergillus*, it is difficult to distinguish subgenus of *Penicillium* from each other. Although many species of *Penicillium* (e.g., *P. cyclopium*, *P. viridicatum*, *P. chrysogenum*) have been reported as OTA producers, they were classified as *P. viridicatum* in genus [15]. At present, the two widely known and accepted OTA producers of *Penicillium* are *P. verrucosum* and *P. nordicum* [15,16]. However, species of *Penicillium* are often isolated from meat and its products, while species of *Aspergillus* are more likely to infect plants and then produce OTA during their pre-harvest and postharvest processes [14]. In addition to grains and coffee, OTA is often found in grapes and red peppers, especially in grapes and their derivative products [17]. The most important OTA-producing fungi in grapes and wine are *A. niger* and *A. carbonarius*. The major pathogenic fungi that can produce OTA are summarized in Figure 2.

Grapes are more likely to be infected by OTA-producing fungi than a variety of fruits and vegetables. Since the first report on the occurrence of OTA in wines and grape juices by Zimmerli and Dick in 1996, many reports have studied OTA contamination in grapes and their products worldwide [18]. The differences in the type of grape products, processing technology, grape varieties and origin of raw material always cause significant differences in the content of OTA [19]. Silva et al. studied 100 wine samples purchased from different Portuguese retail outlets, and 4% of red wine and 1% of white wine were contaminated with OTA, which were produced in different region [20]. Kochman et al. analyzed OTA in red wine from Poland, Spain and France, but unexpectedly, the OTA content in all of the samples significantly exceeded the maximum limit established of 2 µg/L (EU, 2006); the average concentration of OTA even reached 6.629 µg/L in Polish wines, 6.848 µg/L in Spanish wines and 6.711 µg/L in French wines [21]. In China, the maximum concentration of OTA in red wine was 5.65 μg/L [22]. Another study showed a frequency of contamination of 85% (35 out of 41) in American wines with a highest level of approximately 8.6 ± 4.8 µg/L [23]. However, the same research found that samples of OTA content exceeding 1 µg/L were 54.8% in red wine, 40.0% in white wines, 58.1% in dry wine and 30% in sweet wines. OTA levels in red wines are always higher than that in rosés and white wines, because for red wine making the maceration is an important and necessarily step. Since the skins of grapes were the most contaminated tissue of grapes, the maceration facilitated OTA going from the skin to the must, resulting in an increase in toxin content [19].

Grape juices exposed to OTA have been found in different regions of the world. According to previous reports, Mehri et al. investigated the prevalence and concentration of OTA and estimated that the global pooled prevalence of OTA in grape juice was 36% [24]. In Germany, only 7/38 white grape juices were low in OTA content (less than 10 ng/L) and in other samples were 10–1300 ng/L, while 65/73 red grape juice contained OTA between 10–5300 ng/L [25]. In Poland, 26.7% (16/66) of grape beverages tested positive for OTA at the highest value of 0.7 µg/kg [26]. Wei et al. analyzed 41 grape juices and found that all of the samples were exposed to OTA, the incidence of positives was 100% and the OTA concentration of six grape juice samples was between 0.1 and 0.2 μg/kg [27]. In Iran, it was found that the OTA level of 39 out of 70 samples (55.7%) was higher than 2 µg/L, and the upper bound mean was 2.6 μg/L [28]. A total of 1401 fruit juice samples that were produced in Argentina from 2005 to 2013 were evaluated, confirming the identity of only 22 OTA-positive juices, and the concentration of OTA was from 0.03 to 3.6 µg/L [29]. In conclusion, most of the above reports with high OTA concentration were before 2000, and OTA-positive juices seemed to decrease significantly after 2000. The changes of OTA exposition rate and maximum concentration seemed to be related to the progress of OTA detection technology and the strict regulatory limit on OTA in the market.

Although there was a small amount of research about OTA focusing on fruits, vegetables and their processed products other than grapes, a few recent reports of OTA in fruits and vegetables are summarized in Table 1. Sanzani et al. isolated OTA-producing fungi species of *A. westerdijkiae*, *A. ochraceus* and *A. occultus* from tomato; however, the OTA content in the tomato infected by these fungi was not determined in this study [30]. One study from China analyzed the capacity of two *Penicillium* strains to produce OTA in strawberries. OTA was detected in *Penicillium* fermentation broth (2.22 μg/L), but OTA was not detected in spoilage strawberries [31]. However, OTA was found in dried fruits such as figs, apricots, dates, mulberries and raisins [32,33,34,35,36,37,38,39,40,41], though the OTA concentration in dried apricots and dates was much lower compared with dried figs and raisin (Table 1). The difference of OTA content was related to many factors, including the species of fungi, the sugar content, pH, temperature and water activity of the host [42]. Fruits with high sugar levels were conducive to mold growth and induce OTA production, and heat treatment or forced air was easier for eliminating toxigenic fungi than natural drying, so as to reduce the OTA contamination [43].

OTA-contaminated foods, especially plant foods, were related to the occurrence of many diseases in animals and humans, and it was a potential health threat with nephrotoxicity, hepatotoxicity, teratogenicity, immunotoxicity and neurotoxicity [43]. More and more research results indicated that even a very low level of OTA intake will accumulate in the human body [44,45]. The acute toxicity of OTA showed wide variability, which is related to animal sex, species and sample size. The acute dietary 96 h LC_50_ of OTA for sea bass was 9.23 mg/kg BW [46], for rats and mice it was about 46–58 mg/kg BW and that for pigs, cats, rabbits and dogs was 0.2–1 mg/kg BW [47], while LC_50_ for primates and humans was not obtained. However, it has been found that the pathogenesis of OTA is mostly chronic and subchronic toxicity, and the major mechanisms of OTA toxicity were the inhibition of protein synthesis, promotion of membrane peroxidation, disruption of calcium homeostasis, inhibition of mitochondrial respiration and DNA damage [48]. OTA contamination in food has been reported to induce hepatocellular tumors (well differentiated trabecular adenomas), renal cell tumors (renal cystic adenomas and solid renal tumors) and proliferative liver nodules [49]. Therefore, the International Agency for Research on Cancer (IARC) has classified it as an IIB group [50]. Erceg et al. found that OTA produces mild cytotoxic effects in HESCs by inhibiting cell attachment, survival and proliferation [43]. OTA administration induced renal injury of mice and increased histological injury, renal fibrosis molecules and activated the NOD-like receptor protein 3 inflammasome and induced pyroptosis [51,52]. As a neurotoxin, many neurodegenerative diseases, such as Parkinson’s disease and Alzheimer’s disease, are related to the apoptosis of nerve cells and the obstruction of nerve tissue regeneration caused by OTA [53].

### 2.2. Patulin (PAT)

PAT is an α, β-unsaturated γ -lactone, which was first isolated and detected from *Penicillium griseofulvum* in 1943 [54]. Early studies found that patulin has broad-spectrum antimicrobial activity and can also even inhibit the growth of more than 70 kinds of bacteria, but after the 1960s it was reclassified as a mycotoxin [55,56]. PAT was well known as a secondary metabolite produced by *Penicillium*, *Aspergillus*, *Byssochlomys* and *Pacelomyces* (Figure 2) [57]. It was reported that more than 60 species fungi belonging to 30 genera can produce PAT, mainly including 13 *Penicillium* genus and 4 *Aspergillus* genus [1]. The specific PAT pathogens were *P. expansum*, *P. patulin*, *P. claviforme*, *P. novaezeelandiae*, *P. lapiclosum*, *P. granulatum*, *P. cyclopium*, *P. chrysogenum, P. roguefooti*, *A. clavatu*, *A. Nivea*, *A. terreu*, *A. giganteus*, *Byssochlamys nivea*, *Brachypsectra fulva* and *Paecilomyces saturatus* [1,58]. Among them, *P. expansum* and *P. patulin* have an enormous capacity for PAT production in fruits and vegetables and were considered as wound pathogens.

The contamination of PAT is mainly derived from apples, pears, apricots, peaches, blueberries, plums, strawberries and their products, such as juice and jam [59]. PAT has become an important index to judge the quality and safety of apple juice. At present, more than 100 countries and regions in the world have formulated the maximum content limit of patulin in fruit juice and other processed products. China, the United States (U.S.) and the European Union (EU) have set a maximum limit of PAT in fruit juice at 50 µg/L, and the World Health Organization (WHO) and Food and Agriculture Organization (FAO) recommend that the daily intake of PAT per body weight should not exceed 0.4 μg/kg [58,60]. PAT in fruits is mainly caused by diseases such as blue mold rot induced by *P. expansum*, which is the major postharvest disease and problem for apples and apple juice [61]. *P. expansum* often infects fruits through wounds and produces toxins during the transportation and storage periods. As a lipid soluble small molecule, PAT has strong diffusion ability, which can spread into the surrounding healthy pulp tissue. When the rotten fruit is used as raw material to produce fruit juice and other products, it is difficult to eliminate the PAT contamination through conventional food processing due to its stable chemical properties. Thus, PAT will not disappear with product processing but will go further into the fruit products [1]. Hammami et al. detected 45 apples and apple products and found that the PAT levels of 5 out of 12 fresh apples ranged from 3.85 to 17.35 µg/kg, and 100% of the apple juice was contaminated by PAT with an average content of 35.37 µg/kg and 5% higher than 50 µg/kg (EU limit). PAT was found in all baby apple juices, ranging from 7.7–61.3 µg/kg [62]. In Tunisia, 11 out of 30 apple juices presented a contamination of PAT, while 12/30 were contaminated in mixed juices, and the average contents of PAT were 80 and 55 µg/kg, respectively [63]. Other research has also showed that the percentage of PAT-positive samples was 80% in concentrated juice, 64% in apple juice, 33% in apple jam, 50% in mixed juice and 20% in compote, with average content levels of 158.1 µg/L, 45.7 µg/L, 302 µg/L, 28.5 µg/L and 32.3 µg/L, respectively [64].

Some studies have suggested that PAT is difficult to generate in fresh fruits and vegetables. However, due to the wide range of hosts of *P. expansum*, once *P. expansum* infects the appropriate host, conidia will continue to infect the hosts, resulting in brown spots, more extensive rotten and accompanied by the biosynthesis of PAT [58]. The contamination of PAT was mainly found in apples, pears and their products, which has become an important quality and safety evaluation index of apple juice [59,62]. In recent years, more and more reports have detected PAT occurrence in fresh fruits and vegetables. Although, samples from Belgium and Argentina showed that the proportion of PAT-positive samples was low, and the content of PAT in most samples was lower than 50 µg/L [29,65]. In Pakistan, the positive levels of PAT were relatively high, with the content exceeding the European maximum limit [66]. It was reported that PAT content in the rotten parts of fruits infected by fungi was as the high as 1000 µg/kg, and PAT in the whole fruit batch was 21–746 µg/kg, while PAT in fruit products made from rotten fruits reached 0.79–140 μg/kg [3,67]. However, another study showed that the PAT-positive percentage in fresh tomatoes was 10.8%, but none of the 173 tomato-derived products from different countries tested positive [65]. The obtained levels of PAT in other fruits and vegetables other than apples are list in Table 2.

A large number of studies about the negative effects of PAT on the human body have been carried out in the past 50 years. The results have indicated that PAT has acute, chronic and cellular-level toxic effects. The symptoms of acute PAT poisoning are agitation, spasms, edema, convulsions, dyspnea, pulmonary congestion, ulceration, hyperemia and distension of the gastrointestinal [70]. In rodents, the LD_50_ of PAT ranged from 0.1 to 295 mg/kg BW/day, but in poultry and monkeys it was 0.1 and 0.5 mg/kg BW/day [60]. In terms of chronic toxic properties, patulin was reported as a mutagen, carcinogen and teratogen, and these effects often accompanied each other [58]. The toxicology of PAT is concentrated on the oxidative damage and the oxidative stress-induced apoptosis via the intrinsic mitochondrial pathway [60,71]. PAT treatment decreased the activities of antioxidant enzymes and increased the level of ROS in mouse kidney [72]. The inhibition of mitosis by PAT is always accompanied by binuclear cell formation, DNA synthesis damage and chromosomal disorder, which are the main causes of PAT genotoxicity [73]. Chromosome aberrations and gene mutations induced by PAT were found in different cells (C3H mouse mammary carcinoma cell line, V79 cells, FM3A cells and mouse lymphoma L5178Y cells) [70]. PAT may disrupt the composition of the gut microbiota and damage the intestinal barrier, and it has been proven to be related to different intestinal diseases, such as endotoxemia, inflammation, intestinal lesions and endotoxemia [60,74].

### 2.3. Alternaria Toxins (ATs)

*Alternaria* is a notorious pathogen of fruits and vegetables, which can infect tomatoes, apples, citrus and olives and grows at low temperatures, even at −3 °C during storage and transportation [75]. Since the first Alternaria toxins were isolated from *Alternaria kikuchiana* by Tanaka in 1933, more than 70 ATs have been reported, but only 30 ATs with toxic effects on animals have been reported [76,77]. ATs can be divided into four major categories according to their chemical structure and properties: ① dibenzopyrones and related compounds mainly including alternariol (AOH), alternariol monomethyl ether (AME) and alternanene (ALT); ② tetramic acids mainly including tenuazonic acid (TeA) and its isomer iso-TeA; ③ perylene derivative, mainly including alterotoxin I (ATX-I), alterotoxin II (ATX-II) and alterotoxin III (ATX-III); ④ other structures, mainly including tentoxin (TEN) [78]. TeA, AOH and AME are the most common non-host-specific toxins in fruits and vegetables. ATs have frequently been associated with *Alternaria* isolates, the major mycotoxin-producing species was considered to be *A. alternata* and other *Alternaria* species: *A. arborescens*, *A. blumeae, A. tenuissima*, *A. tenuissima*, *A. arborescens*, *A. longipes*, *A. radicina*, *A. dauci*, *A. infectoria*, etc. (Figure 2) [79].

Tomato is an economically important crop that is not only consumed as a fresh crop, but it is also the major ingredient of many processed foods. *Alternaria* causes tomato black spots and always accumulates ATs, resulting in serious economic losses. At present, the percentage of Ats-positive samples in tomato and its products is relatively high, especially that of TeA [80]. As early as 1981, Stinson et al. investigated tomatoes, apples, oranges and lemons infected by *Alternaria* in the U.S. market and found that the main ATs in tomatoes was TeA, with the highest content of 139 mg/kg and positive rate of 57.9%; moreover, small amounts of AOH, AME and ALT were detected [81]. TeA was found in all of the tomato concentrates, 78–100% of tomato sauce samples, 80% of pastes and 50–100% juices, and the maximum concentration of TeA was 100–462 µg/kg. In addition, the detected percentages of AOH, AME and TEN in tomato product samples were 28–86%, 20–78% and 21–64%, respectively [82]. Hickert et al. analyzed nine Alternaria toxins in Germany and found the frequencies of contamination of AOH, AME and TeA in tomato products was 70.6%, 79.4% and 91.2%, and the average amounts were 13.0, 2.5 and 200.0 µg/kg, respectively [83]. In China, it was found that the TeA, AOH, TEN and AME levels in 31 ketchup samples were 10.2–1787 (100%), 2.5–300 (45.2%),1.53–15.8 μg/kg (83.9%) and 0.32–8 μg/kg (90.3%), and only nine tomato juice samples were found without AOH, but TeA, TEN and AME-positive juices were 100.0%, 33.3% and 77.8%, and the concentrations were 7.4–278, 1.85–5.7 and 0.2–5.8 μg/kg [84]. The European Food Safety Authority (EFSA) proposed the threshold for toxicological concern (TTC) method to assess the potential risk of ATs in food to human health according to ATs levels in different foods from 2010–2015 [85]. The upper bound of AOH was 17.1 mg/kg in tomato puree, 17.4 mg/kg in tomato sauce and 17.4 mg/kg sun-dried tomatoes. The high levels of TeA reported were 351 mg/kg in dried tomato soup, 233 mg/kg in sun-dried tomatoes, 212 mg/kg in tomato puree and 54 mg/kg in fresh tomatoes, tomato sauce, tomato ketchup and tomato juice [86].

Fruits, vegetables and their products are rich in nutrients, and they are easily infected by *Alternaria* under suitable conditions. Tournas et al. studied the ability of *A. alternata* to produce AME and AOH at different temperatures, which showed that grapes and apples could produce AME and AOH when stored at a low temperature, and the toxin gradually accumulated with the extension of storage time [87]. The main ATs in apples are AOH and AME, followed by ATX-I and small amounts of ALT and TEA [80,88]. The levels of the five ATs were screened in 11 fresh apples and 7 apple juices, but none were detected [88]. AOH, AME, TeA and ALT were found in different parts of apple, and the proportion of TeA-positive samples was the highest, followed by ALT, AOH and AME. The positive percentages of TeA in the rotten part, junction of disease, and even in the healthy part were 66.35%, 42.31% and 5.77, and those of ALT were 52.88%, 47.17% and 2.88%, respectively. The contamination of AOH and AME was less serious [9]. Some research found the none of AOH and AME tested positive in grape juice, carrot juice and vegetable juice, but AOH and AME in wine were in higher levels [89,90]. The contamination of ATs in fruits, vegetables and their products in recent years is shown in Table 3.

Most ATs have low acute toxicity. However, some previous research has suggested that the most acutely toxic molecule in the group of ATs is TeA [88,95]. TeA is the only ATs listed in the Registry of Toxic Effects of Chemical Substances by the National Institute for Occupational Safety and Health [96]. In female and male mice, the LD_50_ of TeA was 81 and 186 mg/kg, respectively, and in rats it ranged from 168 to 180 mg/kg [97]. TeA has been recently characterized as responsible for dizziness, salivation and vomiting, followed by tachycardia, massive bleeding of the esophagus and gastrointestinal tract, circulatory failure and motor dysfunction [98]. Based on in vitro toxicological studies using mammalian and bacterial cells, it was found that AME and AOH have genotoxicity, carcinogenicity, cytotoxicity and mutagenicity [99]. There is a synergistic effect between AOH and AME, and the toxic effects of AOH or AME on HeLa cells are weaker than that of their mixture. Fehr et al. believed that the toxic mechanisms of AOH and AME are to inhibit the activity of DNA topoisomerase II-alpha isoform and cause cell DNA helix deformation [100]. A high concentration of AOH involving DNA polymerase β mutation and overexpression of DNA polymerase ß causes genetic instability and tumors [80]. In addition, AOH and AME are also related to protein synthesis inhibition, sphingolipid metabolism disorder and photosynthetic phosphorylation [80,101]. Alternaria toxins in cereal grains have been suggested to have a role in the high levels of human esophageal cancer in South Africa and in the Shanxi province of China. Thus, the TTC value of AME and AOH was set to 2.5 ng/kg of body weight per day, and for TEN and TeA it was 1500 ng/kg per day [102].

### 2.4. Trichothecenes (TCs)

Trichothecenes are a class of sesquiterpenoid mycotoxins with a double bond at C_9,10_, and an epoxide ring at C_12, 13_ [82]. According to their diverse structures, more than 200 different TCs have been subdivided into four basic categories: ① Type A has hydroxyl (-OH) or ester (—COOR) at C-8, such as HT-2 toxin, T-2 toxin, diacetoxyscirpenol (DAS), 15-monoacetoxyscirpenol (MAS) and neosolaniol (NEO). ② Type B has carbonyl (—C=O) at C-8, such as deoxynivalenol (DON), 3-acetyl- deoxynivalenol (3-ADON), 15-acetyl-deoxynivalenol (15-ADON), nivalenol (NIV) and fusarenon X (Fus-X). ③ Type C has a second epoxy group on C-7, C-8, C-9 and C-10, such as baccharin and crotocin. ④ Type D contains a single large ring structure at C-4 and C-15, such as satratoxin and roridin [102]. The most common and toxic groups are type A and B, especially DON, also known as vomitoxin [103]. TCs are produced by different fungal species of Fusarium, Trichoderma, Myrothecium, Trichothecium, Cephalosporium, Verticillium, Verticimonosporium and Stachybotrys [104]. Generally, low temperature, high humidity and acidic pH were conducive to the growth of TCs-producing fungi, and the TCs-producing capability was also enhanced [105].

TCs are not only found in cereals (wheat, barley and corn), they also endanger cash crops (potato, muskmelon and apple), as well as livestock products (meat, milk and eggs). Among fruits and vegetables, TCs are widely tested positive for in the dry rot of potato tubers, fusarium rot of muskmelon, core rot of apples and other fruits and vegetables [106]. Xue et al. detected the presence of 3-ADON, Fus-X, T-2 and DAS in the dry rot of potato tubers inoculated with *F. sulphureum*, *F. sambucinum* and *F. solani* and suggested that TCs exist not only in lesions but also in adjacent asymptomatic tissues, and the concentrations of these four toxins were much higher than the maximum level established by the European Union [107]. The maximum permissible limits of DON were 750 µg/kg in flour and 500 µg/kg in breads. Moreover, EU legislations set the maximum residue limits of ZEN, which was 100–200 µg/kg in unprocessed cereals, 75 µg/kg in processed cereals, 20 µg/kg in processed cereal foods and 50 µg/kg in cereal snacks, accordingly [82]. Zhang et al. detected 20 types of mycotoxins in grapes and wines and found 6% of grapes and 7% of wine were detected to be ZEN-positive with concentrations ranging from 0.29–0.36 µg/Kg to <LOQ-1.85 µg/Kg, respectively [108]. The levels of T-2 toxin in the core rot lesions of apple were in the range 7.1–128.4 µg/kg, and NEO was 14.69 μg/kg [109]. The same research group found that NEO accumulated in muskmelon fruits inoculated with *F. sulphureum,* and postharvest ozone treatment or acetylsalicylic acid treatment effectively reduced the concentration of NEO [110,111]. At present, the research of TCs in fruits and vegetables is still in the initial stage, and there are few relevant reports, but that does not mean the toxins will not accumulate in fruits and vegetables. The contamination of the TCs to fruits and vegetables is worthy of extensive and deep research in the future.

For type A and type B, a ring opening can reduce the toxicity of TCs and turn them into low-toxicity or even non-toxic products [112]. The main toxicity of TCs is strong inhibition of protein and nucleic acid synthesis in the body, which results in immunosuppression in human and animal health [6]. In addition, the toxic effect of TCs on humans and animals was related to animal species, age, dosage and the type of TCs. The oral acute LD_50_ of T-2 for mammals and poultry was in the range of 4–10 mg/kg [113]. After oral intake of T-2 in animal diet, it can be absorbed in the digestive tract, which shows toxic effects on the digestive system, causing damage to digestive tract mucosa and finally reducing the absorption rate of nutrients, showing symptoms such as vomiting, diarrhea and extensive visceral bleeding [114,115]. For humans, as a lipophilic substance T-2 easily penetrates into the skin and causes skin irritation at low doses, cell membrane damage at high doses and apoptosis of lymph glands and hematopoietic cells [116,117]. T-2 with strong cytotoxicity can be quickly absorbed by the digestive system and enters the liver, other organs and muscle tissues 3–4 h later. Human feeding leucocyte deficiency (ATA) has also been found to be related to the consumption of T-2 toxin, because in vivo T-2 toxin can affect human hematopoietic stem cells [118].

The toxicity of DON was lower than that of T-2, but its contamination was more extensive. Consuming DON-containing food will cause headaches, nausea, abdominal pain, anemia and decreased immunity, and long-time consumption will increase the risk of carcinogenesis, teratogenesis, toxic renal damage, reproductive disorder and immunosuppression [119]. The cytotoxicity of DON is mainly manifested by a C12,13-epoxy group, which can act on bone marrow hematopoietic cells, lymphocytes and blymphocytes to produce cellular immunotoxicity and cause programmed cell death. DON can also destroy the structure of ribosome to damage its function and inhibit the active center of peptidyl transferase located on the 60S subunit of ribosome to affect the synthesis of protein in cells [120]. According to the research of long-term consumption of DON-contaminated food, it was shown that DON can affect human hematopoietic stem cells and result in human esophageal cancer [121]. Pigs are highly sensitive to DON, which causes mycosis and porcine leukocyte deficiency in Asian and European countries [122]. When the DON content in pig feed exceeded 1 mg/kg, the feed intake of the pigs decreased significantly and was occasionally accompanied by vomiting. The toxic mechanism of low-dose DON on pigs was mainly linked to the inhibition of immune-related genes, which leads to a series of symptoms [123]. However, cattle, sheep and adult chickens and ducks would eat food with DON, because microorganisms in rumen can quickly convert DON into epoxy-DON, which reduces the toxicity [124].

## 3. Determination Technique of Mycotoxins in Fruits and Vegetables

**Figure 3:**
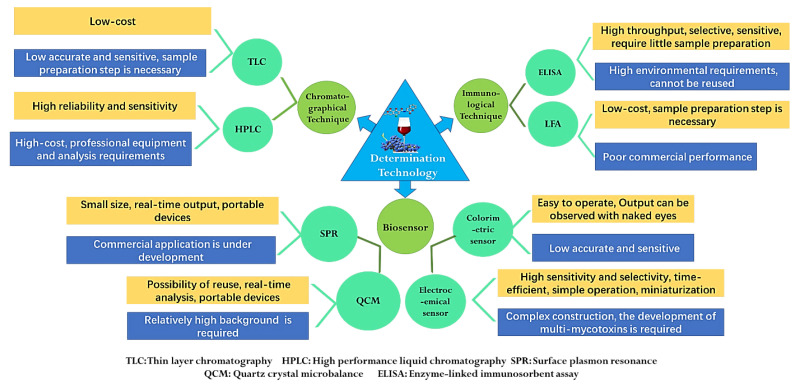
Scheme for mycotoxin determination technologies.

The strict maximum content limits of mycotoxin all over the world have greatly promoted the development of mycotoxin detection technology. In addition, the levels of mycotoxins in fruits and vegetables are very low, which puts forward higher technical requirements for the accuracy and sensitivity of the detection. The food matrix has complex components and trace amounts of mycotoxins, so it is difficult to analyze the content of mycotoxins directly. Therefore, except enzyme-linked immunosorbent assay, most detection methods require efficient sample pretreatment techniques for the separation and enrichment of mycotoxins [12]. Generally, for mycotoxins with different chemical structures and matrices, it is necessary to select correct sample pretreatment or clean-up methods. The common extraction methods include liquid–liquid extraction, solid phase extraction (SPE), supercritical fluid extraction (SFE), gel permeation chromatography (GPC) and immunoaffinity cleanup (IAC) [2]. At present, mycotoxin detection methods mainly include chromatographical techniques, immunological techniques and biosensors, which can derive many different technologies according to the pretreatment methods, detection methods and detection principles [125]. The quantitative procedures for mycotoxins have developed rapidly and have different advantages and disadvantages. However, mycotoxins always have different chemical structures and properties, so it is difficult to analyze multi-mycotoxins at the same time, which is a common problem that must be solved [126]. The characteristics of different technologies for mycotoxin determination are shown in Figure 3.

### 3.1. Chromatographical Technique

Techniques based on chromatography include thin-layer chromatography (TLC), high-performance liquid chromatography (HPLC) and gas chromatography (GC), which are always coupled with ultraviolet (UV), diode array (DAD), fluorescence (FD), diode array (DAD) and mass spectrometry (MS) detector and use immunoaffinity clean-up as pretreatment [127]. HPLC-FD has been utilized for the detection of ZEA, OTA and DON. HPLC-PDA was reported for analysis of ATs, and HPLC-PDA-FD was applied for DON, OTA and ZEA determination [37,64,93]. During the mycotoxin analysis, because chromatography can provide accurate and reliable results, many organizations around the world, such as the International Organization for Standardization (ISO), the Association of Official Analytical Chemists (AOAC) and the European Committee for Standardization (CEN), mostly choose chromatography as the standard method for mycotoxin detection [128,129,130]. As early as 1993, the ISO issued the detection method standard of practical HPLC-UV for the detection of PAT in apple juice [128]. The CEN set standard analysis methods of PAT in apple products and OTA in grape products and dried fruits. These standards adopt liquid–liquid distribution purification, solid-phase extraction column purification or immunoaffinity column combined with HPLC chromatography. The AOAC also formulated three PAT test standards, including the TLC method and HPLC and IAC-HPLC for OTA determination [131]. Although chromatography is widely accepted by international organizations, they generally have the limits of being time-consuming, needing a professional tester and expensive laboratory equipment (Figure 3).

Liquid chromatography–tandem mass spectrometry (LC–MS/MS) has the advantages of the chromatographic separation and molecular identification of mass spectrometry, which has become the main method for multi-mycotoxins analysis in recent years [80]. Based on isotope dilution, five ATs in tomato-based samples were quantified by LC–MS/MS, with a limit of quantification (LOD) lower than 1.0 µg/kg and a recovery of 52.7–111%. The possible matrix effects were compensated by the corresponding isotopically labeled internal standards [132]. In Belgium, UPLC–MS/MS was applied to quantitate six different ATs in 129 commercial fruit and vegetable juices and tomato products with the preparation and extraction protocol of QuEChERS (quick, easy, cheap, effective, rugged and safe). The quantitation limits (0.7–5.7 µg/kg), repeatability (RSDr < 15.7%), reproducibility (RSDR < 29 17.9%) and recovery (87.0–110.6%) were acceptable [98]. AOH and AFB1 were extracted from fruit juice and fruit juice/milk beverages and then controlled by dispersive liquid–liquid microextraction (DLLME) and determined by HPLC–MS/MS-IT [133]. A rapid solid-phase extraction (SPE) method to the LC–MS/MS system was developed to determine nine mycotoxins in five types of commonly used betel nut products, with an LOQ of 0.25–10 μg/L, an LOD of 5–20 μg/L and recovery in the range of 70.1–113.9% [134]. The interference of the matrix in the multiple detection of mycotoxins has been greatly reduced, and the detection sensitivity and accuracy of LC–MS/MS has been improved, but the detection cost was still high. Developing a fast, low-cost method suitable for on-site detection of mycotoxins is the research hotspot of the LC–MS/MS method in the future.

### 3.2. Biosensor Technique

#### 3.2.1. Antibody and Aptamer

The biosensor technique has become a low-cost and fast alternative to traditional chromatographic methods in the field of mycotoxin analysis. The biosensor technique refers to the technique of using biological substances (such as antibodies, antigens, aptamers, peptides, cells, etc.) as recognition elements to transform biochemical reactions into quantitative physical and chemical signals through appropriate energy exchangers (electrochemical, piezoelectric, spectral, thermal and surface plasmon resonance) [135]. Biosensors can be divided into four groups based on the physicochemical properties of mycotoxins and the type of transduction: optical sensors (SPR), piezoelectric sensors (QCM), colorimetric and electrochemical sensors [136]. The antibody is identified through an in vivo process, induced by the biological-system-producing immune response to the target molecule in animals, and is obtained by further screening [131]. Therefore, the antibody has high specificity and affinity for the target, and the antibody-based immunochemical assays often have high selectivity, sensitivity and a low detection limit. Up to now, many mycotoxin antibodies have been screened and gradually commercialized, such as AFB1, OTA, DON, ZEN, PAT, etc. [137]. As a chemically synthesized single-stranded nucleic acid, an aptamer capable of binding and highly specific recognition to the target was obtained in vitro by systematic evolution of the ligand by the exponential enrichment process (SELEX) [126]. So far, six mycotoxin aptamers have been screened, which involved OTA, PAT, ZEN, FB1 and T-2 [138], and some sequences of these aptamers are shown in Table 4. Up to now, both antibodies and aptamers have been widely used as mycotoxin recognition elements and constructed the different types of sensors mentioned above.

#### 3.2.2. SPR, QCM and Colorimetric Sensor

A surface plasmon resonance sensor is always applied to characterize and quantify the biomolecular interactions. A surface plasmon is excited in the refractive index at an interface between media of different refractive indices, and the changes are measured. That means that the changes in the mass concentration of material on the surface are crucial to the sensitivity of SPR sensors [126,141]. Zhu et al. reported that an SPR biosensor involved an anti-OTA aptamer by immobilized a sensor chip, and that was used to quantitatively analyze OTA with a lower detection limit of 0.005 μg/L, and a recovery of 86.5–116.5% in red wine. The principle is that the biotin–aptamer can be captured through streptavidin–biotin interaction [141]. A multi-mycotoxins detection assay based on an SPR instrument and two biosensor chips (one for DON, ZEN and T-2 toxin and the other for OTA, FUMB1 and AFB1) was developed, which was modified by ovalbumin (OVA) conjugates of mycotoxins. The SPR response was recorded when antibodies mixed with samples were added to a chip. The LODs of DON, ZEN, T-2, OTA, FUMB1 and AFB1 were 26, 6, 0.6, 3, 2 and 0.6 µg/kg, respectively [126]. A competitive inhibition iSPR biosensor was prepared through the competition between the immobilized mycotoxins on a nanostructured iSPR chip and the free mycotoxins in the solution. The LODs were 17 μg/L for DON and 7 μg/L for OTA [142]. Although the SPR biosensor has been widely studied in academic research, in commercial application the professional requirements of technology and data analysis should be optimized, and cheaper labeling reagents should be prepared and applied to cut costs.

The quartz crystal microbalance is a biosensor platform that has the component of a thin quartz crystal disk in between two gold electrodes, one of which is typically coated with an antigen conjugate to sense mycotoxins [143]. A label-free QCM-based immunosensor was developed by direct immobilization of OTA to amine-bearing sensor surfaces using 1-ethyl-3-(3-dimethylaminopropyl) carbodiimide (EDC)/N-hydroxysuccinimide (NHS) chemistry, which can measure OTA in red wine with a range of 17.2–200 μg/L [144]. Karczmarczyk et al. applied a secondary antibody conjugated to gold nanoparticles to amplify the signal of a QCM-based sensor, which has a lower LOD of 0.16 μg/L for OTA determination in red wine [145]. However, commercialization of a QCM biosensor is limited by the thickness of the quartz plates, but thinner materials with higher mass sensitivities are quite expensive.

Colorimetric sensors based on aptamer have simplicity, rapidity, lower cost and are more suitable for on-site detection. A T-2 colorimetric aptasensor was developed based on the aggregation of gold nanoparticles (AuNPs) in the presence of T-2. When an aptamer bound with T-2 would release AuNPs and make the color change from red to purple blue, the LOD of this method was 57.8 pg/mL [146]. Yang et al. also used the stability against the salt-induced aggregation effect of AuNPs in the presence of OTA’s aptamer to build a colorimetric sensor and proved that the aptamers form G-quadruplexes after binding OTA [147]. He et al. proposed an innovative colorimetric method based on enzyme-induced gold nanoparticle aggregation, which can monitor OTA concentrations in red wine and grape juice. OTA aptamer, biotinylated cDNA and SA–ALP were modified on DNA-ALP-immobilized MBs. In the presence of OTA, the binding of OTA with aptamers switched the aptamer structure from an aptamer–cDNA duplex to an aptamer–OTA complex, and then ALP-cDNA was liberated and converted AAP to AA. MnO_2_ was reduced by AA and produced Mn^2+^, which leads to aggregation of AuNPs, and a color change from brownish red to blue was detected [148]. Due to the dependence of the chemical properties of mycotoxin, colorimetric sensing is vulnerable to the interference of the environment, which affects the accuracy of the detection results. By improving the stability of gold nanoparticles, the accuracy of the detection method would be increased based on the color change of its surface plasma corresponding to the spectral shift.

#### 3.2.3. Electrochemical Sensor

Electrochemical sensing can be divided into label and label-free approaches, most of which apply cyclic voltammetry (CV), differential pulse voltammetry (DPV), square wave voltammetry (SWV) and electrochemical impedance spectroscopy (EIS) to monitor the change of current, circuit potential or resistance of the detection system [86]. There are many kinds of substances that can be used to label aptamer sensors, such as radioactive or fluorescent dyes, metal complexes, quantum dots, nanoparticles and enzymes. This label attached to the target molecule, aptamer or antibody always has its own common feature of easy conjugation and detection, but the labeling and immobilization steps are time-consuming and expensive [149]. Some researchers have developed an aptamer sensor for detecting OTA under flow conditions. An automated flow-based aptasensor was utilized for OTA detection with a flow injection system with a detection limit of 0.05 μg/L. Biotin-labeled OTA and free OTA compete to bind the aptamer on the surface of the printed electrode in the central flow cell, which reduces the analysis time [150]. Abnous et al. modified methylene blue on the double-stranded DNA formed by the OTA aptamer and its cDNA. The combination between aptamer and OTA prohibited the binding of cDNA and the aptamer and the release of MB accompanied by the reduction of the signal. The LOD of OTA in grape juice was 58 pM [151]. Based on the good electrical conductivity and large specific surface area of thionine (Thi)-labeled Fe_3_O_4_ nanoparticles (Fe_3_O_4_NPs)/rGO and rigid structure of tetrahedral DNA nanostructures (TDNs), an electrochemical aptasensor for PAT was constructed to analyze PAT in apple juice. Once PAT bound with the aptamer, the aptamer was released from the TDNs-Apt, and Fe_3_O_4_NPs/rGO with Thi was introduced to the electrode surface, resulting in significant Thi signal changes. The proposed aptasensor showed an excellent LOD of 30.4 fg/mL and a recovery in apple juice of 96.9–105.8% [152]. According to most studies of labeled electrochemical sensors, it could be found that voltammetry was the common approach to detect mycotoxins by measuring the variation of the system current.

The label-free electrochemical sensor can directly measure the electroactive substances or the current generated by the oxidation and reduction of probe molecules, which corresponds to the concentration of analyte in the sample. The detection signal of the label-free aptamer sensor is directly related to the interaction between the target and the aptamer, and it can avoid false-positive results and difficulty in the labeling process [149]. A label-free sensor was developed based on anti-PAT-BSA IgG, which was immobilized on the graphene oxide/gold nanocomposite-coated glass carbon electrode (GCE). The combination between anti-PAT-BSA IgG and PAT reduced the spatial hindrance effect of the sensor and resulted in a decrease in the electron transfer resistance. The current changes exhibited a linear relationship with the patulin concentration with an LOD of 5 g/L [153]. Xu et al. modified GCE with black phosphorus nanosheets (BP NSs) and an electrostatic-adsorption-modified PAT aptamer on the surface. The same research also found a wider linear range and LODs could be obtained by modifying AuNPs and thiolated PAT aptamer on the BP NS-GCE [154]. AuNPs and thiolated OTA aptamer were immobilized on the surface of the gold electrode through layer-by-layer self-assembly. This label-free impedimetric aptasensor was applied to quantify the OTA concentration in grapes and their products. The OTA aptamer changed its random coil structure into the antiparallel G-quadruplex conformation in presence of OTA, because the electrostatic and steric repulsion the diffusion of [Fe(CN)_6_]^3−^ into the surface of the electrode was hindered, and the relative normalized electron-transfer resistance values were proportional to the concentration of OTA [155]. In a further study, the same team proved that AuNPs can effectively improve the specific gold surface area and fix more aptamers. When it comes to regaining of the aptasensor, it was found that the modification density of thiolated aptamer was maintained relatively constantly while that of the amino aptamer decreased [156]. Because the sensitivity improvement of the label-free electrochemical mostly depends on the aptamer modification levels, it is extremely significant to screen and prepare novel electrode modified materials that can increase the specific surface area, especially novel nanomaterials. Different from the labeled sensor, EIS is more suitable for monitoring the resistance changes caused by the aptamer structural transformation modified on the electrode surface. In order to promote the development of electrochemical sensors for mycotoxins, the sample pretreatment process and immobilization of molecular recognition elements should be further simplified, and simultaneous detection technologies for multi-mycotoxins analysis need to be further developed.

### 3.3. Immunological Technique

As an important rapid quantification detection technique, immunochemical assays include major examples such enzyme-linked immunosorbent assay (ELISA), dipsticks, flow-through membranes and LFDs. Antibodies and antigens were very effective immunochemical tools that were wildly applied in the mycotoxin detection filed [86]. For example, the principle of ELISA is the interaction of the antigen–antibody complex with the chromogenic substrates and measuring the developed color by spectrophotometry. There are many enzymes applied to replace the immunoassay in combination with a substrate, among them horse radish peroxide (HRP) and alkaline phosphatase (ALP) are widely used [157]. ELISA has been developed for ZEN, AF’s, OTA, T-2 and FUM monitoring with a short incubation period time of 15 min, but most are between 1 and 2 h [152]. Wang et al. designed a hapten of TeA mycotoxin by computer-assisted modeling to produce the highly specific camel polyclonal antibody and established an indirect competitive chemiluminescence enzyme immunoassay (icCLEIA) that has high sensitivity, high specificity and acceptable recoveries for TeA detection in fruit juices [158]. Pei et al. presented a pELISA measurement of OTA based on the competing antigen of OTA-labeled urease, which can hydrolyze urea into ammonia. The ammonia molecules increased the pH, so silver ions were reduced by the formyl group from glucose to generate a silver shell and resulted in the changing of the solution color from blue to brownish red. The LOD of OTA was 40 pg/mL, 15.6- and 14.3-folds lower than those of HRP based ELISA [159]. A sensitive and novel multiplex nano-array based on ELISA has been developed allowing for the simultaneous semi-quantitative analysis of ZEA, T-2 and FUM in around 70 min due to the cross-reactivity profiles of the antibodies used. The conjugates of three mycotoxins were nano-spotted into the single wells of a microplate, and a calibration curve for the concentration was analyzed by a competitive assay format [160]. For ELISA, because this technology mostly depends on the biological activity of antibodies and antigens, they are very sensitive to temperature and pH variations and undergo irreversible denaturation. The application of regenerable and stable cognitive elements as a substitute for antibodies is an important means to improve the stability and feasibility of this technology.

Lateral flow immunoassays (LFA) take the cellulose or glass fiber as the solid phase and move the sample smoothly and homogenously on the chromatographic material with the help of capillarity. Antibody-labeling tag conjugates are usually used as the labeled reagents that could interact with the pathogens in the moving liquid sample. Visual detection results can be obtained in a short time through direct color markers or enzymatic color reaction [138,161]. LFA can monitor the detection results by the naked eye, which has become an excellent on-site detection technology. The labeling tags mainly include quantum dots, AuNPs and luminescent nanoparticles [143]. OTA in grape juice and wine was determined by a silver nanoparticle-based fluorescence-quenching lateral flow immunoassay with a competitive format (cLFIA). As background fluorescence signals, Ru(phen)32+-doped silica nanoparticles (RuNPs) were sprayed on the test and control line zones, and its fluorescence quenchers were AgNPs. The proposed method exhibited an acceptable LOD of 0.06 g/L and average recoveries of 88.0–110.0% in red grape wine and 92.0–110.0% in grape juice [162]. A multiplex immunochromatographic test strip based on 25 nm AuNPs was designed for simultaneous detection of FB1, DON and ZEN in 15 min. The different mycotoxin conjugates (FB1–BSA, DON–BSA and ZEN–BSA) and goat anti-mouse IgG were separately spotted onto the NC membranes as three T lines, which dried together with antibody–AuNP conjugates sprayed on the conjugate pad. In the presence of the target mycotoxins, a part of the antibody–AuNP conjugates will react with the respective antigens in the sample pad to competitively inhibit the interaction of the T line and the resulting change of color [163]. Epoxy-functionalized silica-coated QDs showing green, orange and red color were conjugated with anti-ZEN, anti-DON and anti-T-2 mAb for multiplex lateral flow immunoassay (LFIA) preparation. The LFIA can detect ZEN, DON and T-2 toxin in 15 min, and the false-negative rate is lower than 5% [164]. Although the sensitivity and accuracy of LFA have been improved, reducing nonspecific adsorption and avoiding the interference of background fluorescent substances still limit the development of this technology. Research on the modification and immobilization technology of antigens and antibodies on the surface of a microfluidic chip can greatly improve the sensitivity and repeatability of detection.

## 4. Degradation Technique of Mycotoxins in Fruits and Vegetables

Patulin, ochratoxin, Alternaria toxins and trichothecenes are the major mycotoxins in fruits and vegetables, whose contamination is inevitable. These mycotoxins are resistant to heating, and the common heating operation is difficult to remove or degrade. Therefore, developing an efficient removal strategy is a challenge for researchers. Mycotoxin removal involves adsorption and degradation in terms of mechanisms. Adsorption treatment is to remove mycotoxin by applying an absorbent with a large specific surface area. Degradation treatment is to remove mycotoxin by some strategies to destroy the chemical structure of the mycotoxins, especially the toxic group in the chemical structure. Physical, chemical and biological methods are the main strategies to degrade mycotoxins in fruits and vegetables.

### 4.1. Physical Method

#### 4.1.1. Physical Adsorption

Absorption treatment is based on adsorbents with a tremendous specific surface area, which is a widely used physical strategy to remove mycotoxins. Among the many commercial adsorbents used for removal of mycotoxins, activated carbon, macroporous resin and diatomite are the commonly used physical adsorbents. Activated carbon treatment significantly reduces patulin content in apple juice, and more importantly, no significant changes to the nutrition and quality of the apple juice, such as soluble solids content, reducing sugar and total acid, were observed [165]. The composite carbon adsorbent (CCA) can be prepared by absorbing ultrafine activated carbon particles onto granular quartz and packing them in a fixed bed adsorption column that has excellent adsorptive properties such as a huge carbonaceous surface area, excellent bed porosity and higher bulk density, which could efficiently enhance the adsorption rate for patulin in both aqueous solutions and apple juice; however, the appearance and flavor of the apple juice were affected [166]. In fact, some porous chemicals can achieve the same effect as activated carbon. For instance, the contaminated apple juice was treated with 0.5% polyvinylpolypyrrolidone (PVPP) or 0.5% beta-cyclodextrin (β-CD) for 24 h, which not only reduced the patulin level in the apple juice but also decreased the total phenols content, avoiding browning of the apple juice. Moreover, activated carbon is also applied to absorb ochratoxin (OTA) and deoxynivalenol (DON); nevertheless, when activated carbon absorb the two mycotoxins, some nutrient substances were simultaneously absorbed, which markedly influences the quality and nutritional value [167].

Macroporous resin is widely applied to absorb patulin in apple juice, and macroporous resin can be divided into different types according to their source and pore size. Liu et al. compared eight types of macroporous resin of LSA-900B, XDA-600, LS-803, LS-806, LSF-500, HPD-850, DM-2 and DM-3 for adsorption of patulin from apple juice and suggested that LSA-900B indicated the highest absorbent effect (92.55% at 50 °C); more importantly, the quality parameters such as absorbance, color value, light transmittance and turbidity were remarkably improved [168]. XDA-600 macroporous resin also displayed a higher adsorption efficiency for patulin in pear juice at 25 °C, in which the adsorption behavior is suited to the adsorption thermodynamic Freundlich isotherm and line with the quasi-secondary rate equation [169].

Attapulgite is regarded as a green material in the 21st century due to the advantages of extensive resources, low cost, nontoxic nature, environmental friendliness and unique three-dimensional spatial and large specific surface area, which gives attapulgite an unusual adsorption property. Liu et al. screened the optimal adsorption condition, which was 0.0800 g of attapulgite at 40 °C for 22 h based on the Box–Behnken experimental design [170]. Zhang et al. suggested that the attapulgite adsorption process follows the Freundlich isotherms model and the pseudo first-order kinetics model [171]. In fact, the ion exchange and chelation processes were associated with attapulgite adsorption for patulin, which leads to a slight decrease in the contents of total acid, reducing sugar, viscosity, soluble solids and total phenols. The effort attempted to overcome the influence on juice quality by modification and developed a magnetic attapulgite (Fe_3_O_4_@ATP) that was prepared by precipitation and the spreading of Fe_3_O_4_ nanoparticles on attapulgite (ATP). Then, the magnetic adsorbent was used to remove mycotoxins from contaminated peanut oil, and the results suggested that the magnetic adsorbent of Fe_3_O_4_@ATP displayed an excellent removal efficiency of 86.82% for mycotoxin contamination [172].

**Figure 4:**
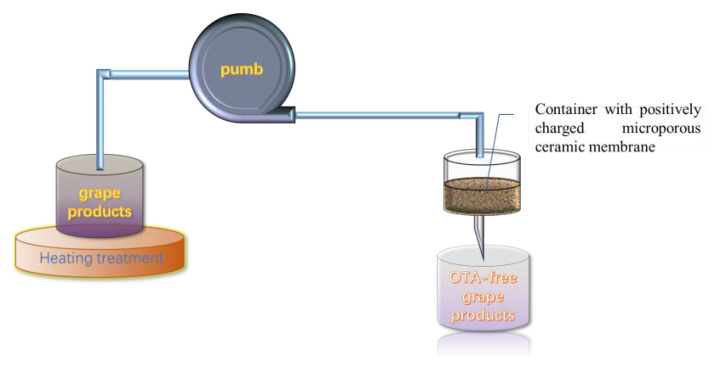
Schematic of the removal device with a nano-MgO/diatomite ceramic membrane.

A microporous ceramic membrane has been employed for the removal toxic or harmful contamination. However, the removal efficiency is not always satisfying. In order to improve the adsorption efficiency, Nan et al. developed a nano-MgO-modified diatomite ceramic membrane (MCM) with a high positive charge and successfully applied it to remove OTA in wine (Figure 4). The main adsorption principle is that the nano-MgO MCM displays a positive charge from pH 2 to 12.8, after dispersing nano-MgO particles on the surface of the ceramic membrane, and OTA is negatively charged. Therefore, it is taking advantage of the electrostatic adsorption between the positively charged nano-MgO MCM and the negatively charged OTA to effectively remove OTA in wine. At the same time, the isotherm adsorption was verified to suit to Langmuir model, with a maximum adsorption capacity of 806 ng/g at 25 °C [173]. More importantly, no significant changes were observed on the quality and flavor of the wines.

Molecular imprinting technology (MIT) has gained more and more attention from researchers due to its broad range of potential application, especially in controlling contamination. Molecularly imprinted polymers (MIP) are a kind of cross-linked polymer, which can bind the target compound with high specificity. MIP could be synthesized with specific recognition sites complementary in shape, size and functional group to template molecules [174]. Amino groups could be introduced onto the silica surface with 3-aminopropyltriethoxysilane, and then azo initiation onto the silica surface was achieved by the reaction of the surface amino groups with 4,4′-azobis(4-cyanopentanoic acid). The synthesized MIP was then performed in the presence of 6-hydroxynicotinic acid as template substitute, functional and cross-linking monomers, and the result showed that the synthesized MIP had specific adsorption qualities for patulin, with a 93.97% adsorption rate and a 0.654 µg/mg adsorption capacity [175]. Similarly, Sun et al. achieved a more effective adsorbent for patulin, Fe_3_O_4_@SiO_2_@CS-GO@MIP, and during the synthesis process, Fe_3_O_4_ was added into the reaction to make the MIP adsorbent magnetic. Finally, chitosan (CS) and SiO_2_ were introduced to improve the biocompatibility, stability and dispersibility of the coated MIP adsorbent, and the results indicated that the proposed adsorbent had an efficient adsorption for patulin in apple juice, with an adsorption capacity of 7.11 mg/g and an adsorption rate of 90% after 24 h of adsorption [176].

#### 4.1.2. Physical Removal and Detoxification

Most mycotoxins are heat-stable and form toxic degradation products. Although several detoxification methods have been developed, only a few have been accepted for practical use. The common treatments for removal or detoxification of mycotoxins include heating, microwaving and irradiation, etc. These treatments can inactivate or degrade mycotoxins by destroying the structure of the mycotoxins.

(1) Heating

In general, mycotoxins are relatively stable during heating, and the usual boiling and autoclaving treatments do not easily destroy the structure [177]. The degree of mycotoxin degradation by heating processes largely depends on factors such as temperature, moisture content and time period. OTA, the most widespread contaminant worldwide, can lead to human carcinogen with its potent nephrotoxicity, and a 90% OTA reduction was achieved when heated at 200–250 °C for a longer treatment period [178]. Alternaria toxins mainly include AOH, AME and TeA, and only a few AOH and AME were degraded when heating at 100 °C for 90 min, while the higher degradation efficiency of AOH and AME could be achieved when heating at 121 °C for 60 min. Similarly, the TeA concentration reduced by 50% after 90 min of heating [179]. Trichothecenes are usually stable at 120 °C; however, trichothecenes will partly decompose when the temperature is above 200 °C [180]. However, overall, heating treatment not only needs more energy but also leads to a lot of nutrient destruction. Therefore, heating treatment is difficult to widely apply to remove and degrade mycotoxins in fruits and vegetables.

(2) Microwave

Microwaves, electromagnetic waves with a certain frequency of 103–105 MHz, can come into contact with contaminants to cause a molecular dipole swing, producing high frequency fluctuation to cause a heat effect. A medium heat microwave treatment of patulin (concentration ranges from 100 to1000 μg/L) for 90 s results in an almost complete degradation of 100%. Microwave-treated patulin in apricot juice for 3 min to leads to a reduction of 39%; however, the degradation efficiency reached 95% after 15 min of microwave treatment, and more importantly, no significant changes to nutrition and flavor were found [181]. Microwave treatment is more suitable for the acidic conditions of apple juice because it can better preserve the critical nutrients of the juice. Therefore, microwaving can be widely employed to sterilize and condense apple juice.

(3) Irradiation

Irradiation contains ionic types (such as, X-rays, γ-rays and ultraviolet light) and nonionic types (such as radio waves, microwaves, infrared radiation and visible light). For ionic irradiation, the structure of mycotoxin will be destroyed even treated at a lower temperature, which is severe harmful for humans. For nonionic irradiation, only when the sample is exposed for a long time to cause an increase in temperature can the mycotoxin structure be destroyed; however, nonionic irradiation will not pose harm or threat to humans. Most mycotoxins can be decomposed into nontoxic or less toxic middle products after exposure to nonionic irradiation. Ionic irradiation displays better effects on liquid food than solid food, and the reason may be that it has weak penetration with solid food. The application of γ-rays significantly reduced mycotoxin contamination in juice due to its high efficiency, quickness and there being no secondary pollution. γ-ray exposure significantly reduced the patulin content in citrus juice, and with the increase of irradiation dose, the degradation effect was improved [182]. When the irradiation dose was 8 kGy, the degradation rate of patulin in apple juice could reach 100%. γ-ray treatment also destroys the OTA structure, when the exposure dose was 6 kGy, and the degradation rate of OTA was 96.2% [183]. Similarly, ^60^Co-γ irradiation has excellent degradation efficiency on TeA, and a lower dose irradiation has more influence on TeA than a higher dose irradiation; more importantly, no significant influence was found on the juice quality such as the amino acids, reducing sugars and titratable acids. However, the chromatic value and clarity of juice were improved [184]. The Food and Drug Administration (FDA) regulations state that γ-ray treatment is safe for humans when the dose is less than 10 kGy. Therefore, γ irradiation treatment is an effective method to remove and degrade mycotoxin from vegetables and fruits.

UV irradiation has been widely used as an effective approach to remove mycotoxins because of its lower secondary pollution and lesser influence on degradation production. UV irradiation showed remarkable degradation efficiency on patulin from apple juice. When the initial concentration of patulin was 500 µg/L, it was almost completely degraded after 90 min of UV-C irradiation of 255 nm at 25 °C, pH 7.0; the same degradation effect could be achieved after 90 min of irradiation when the temperature is increased to 65 °C. However, when the pH was 4.0, the patulin degradation rate reached 100% after 40 min of irradiation [185]. Moreover, different wavelengths of UV-C have distinct degradation efficiencies, which is following that 222 nm > 282 nm > 254 nm. After the treatment of UV-C for 222 nm, the L*, a* and b* values were reduced, as well as the content of vitamin C, and the structure of OTA was broken; similarly, environmental temperature and pH significantly influence the UV degradation of OTA. High temperature accelerates OTA degradation, for instance, and UV-C (255 nm) exposure for 6.5 min at 45 °C achieves the same effect as 12 min at 15 °C [186]. In addition, UV irradiation also has the significant degradation effect on trichothecenes, and the factors such as irradiation time, distance and medium pH significantly influence trichothecene degradation efficiency. The degradation of DON and T-2 has a positive relationship with irradiation time and a negative relationship with irradiation distance, and the degradation efficiency reduces as the medium pH increases [187].

At present, irradiation technology does not have widespread application because of the acceptance by consumers. However, with the development of irradiation technology and the management system of food security is extensively applied in the food industry, as well as the further study of the irradiation degradation of mycotoxins, and the application of irradiation technology to remove mycotoxin will be more widespread.

### 4.2. Chemical Degradation

Treatment with chemicals can efficiently remove mycotoxins from fruits and vegetables and their derivative products by using strong oxidant, acid, base and other chemical substances, which decompose mycotoxins by chemical reactions to destroy the structure of the mycotoxins. Ozone, one kind of strong oxidant, has the ability to oxidize the double bond of mycotoxin structure and is widely applied to degrade patulin, OTA and trichothecene. For instance, the patulin was completely degraded after 7–12 mg/L of ozone treatment for 30 min the ozone almost completely degraded the patulin content from 50–500 μg/L in apple juice and the degradation effect is increasing with the extension of the processing time. Ozone treatment had no significant effects on the juice quality of soluble solids, pH and total acid; however, it obviously decreased the color, malic acid, ascorbic acid and total phenols of the apple juice [188]. OTA was effectively degraded after 50 mg/L of ozone combination electron beam irradiation treatment for 180 s [189]. Ozone treatment also reduced neosolaniol (NEO) content (one kind of trichothecene) from muskmelon fruits. When 1.10 mg/L was ozone-treated for 120 min, the content of NEO from fusarium rot of muskmelon fruit was significantly decreased. The possible involved mechanism is that ozone firstly attacks the C9,10 double bond in the NEO structure by electrophilic addition, leading to the change in oxidation state for the C9,10 double bond, then the remaining NEO are left intact at this stage of the reaction, and the two expected keto aldehydes are generated by isomerization at C11 after formation of the aldehyde at the site of C10 [111]. During the ozone processing of the degradation of mycotoxin from juice, few effects on pH, vitamin C, soluble solids content and color value were found; therefore, ozone is widely used in the food processing industry to remove patulin from juice and improve the safety of juice, which is recognized as an effective, safe and cheap method to remove mycotoxins from fruits and vegetables and their juice products.

Glow discharge plasma (GDP) is a novel electrochemical technology in which plasma is sustained by DC glow discharges between a pointed electrode and surface of liquid electrolyte [190]. The possible mechanism is various active substances, such as hydrogen peroxide (H_2_O_2_) and hydroxyl radicals (·OH), that can be generated during electrode discharge and diffuse into the media solution to oxidize the organic molecules. GDP completely destroys organic molecules and produces CO_2_ and H_2_O. Therefore, GDP is widely used to degrade organic contaminants. Pu et al. developed a GDP reactor and firstly used it to degrade patulin in juice and T-2 toxins in water. When the DC voltage was 550 V and the current range was from 145 to155 mA, GDP effectively degraded patulin by 96.63% in 5 min, and no significant effect on the quality and nutrients of the apple juice were observed. The same degradation efficiency was shown in T-2 toxins after 40 min of GDP treatment, and cytotoxicity tests have shown no toxic effect [191].

In addition, ascorbic acid (vitamin C), sulfur dioxide, thiamine (vitamin B1), vitamin B6 and calcium pantothenate were efficiently employed for degrading mycotoxins. For instance, 482 mg/L of ascorbic acid reduced the patulin content to 30% after 34 d of incubation [192]. Acetic acid, propionic acid and other fatty acids with low carbon chains treated the juice contaminated with OTA, and the OTA content was markedly decreased. The treatment of OTA with sodium hypochlorite and formaldehyde significantly destroyed the OTA structure to obtain a product with less toxicity.

Treatment with chemicals, to some extent, effectively degrades and removes mycotoxins from fruits and vegetables and their derivative products; however, rather than complete degradation, there is formation of the degradation products with toxic residues, which is likely to change the nutritional ingredients of the juice. Therefore, chemical methods are somewhat restricted by juice manufacturers.

### 4.3. Biological Degradation

Biodegradation is considered as one of the promising and reliable approaches for control of mycotoxin contamination. The involved mechanism is to destroy the chemical structure of the mycotoxin by microbiology, or the enzymes secreted by microbiology reacting with the mycotoxin. At present, biological degradation has become a hotspot issue worldwide because of its minimal side effects and it being environmentally friendly. Biological degradation mainly includes bacteria, yeast and enzymes secreted by fungi.

*Acinetobacter* spp., *Lactobacillus* spp., *Bifidobacterium* spp. and *Streptococcus lactis* are the main bacterial strains for removing mycotoxin. Treatment of OTA with *Acinetobacter* spp. was also found to reduce OTA by 80% for 24 h, and OTα was detected as the degradation byproduct of OTA; however, inactivated *Acinetobacter* spp. had no detoxification ability, the involved mechanism was responsible for enzymatic biodegradation and the degradation efficiency decreased with the descent of the OTA concentration [193]. *Lactobacillus*, as one kind of important gastrointestinal probiotic, plays an indispensable role in the detoxification of mycotoxin from fruits and vegetables [194]. Some strains of lactic acid bacteria such as *Lactobacillus* spp., *Bifidobacterium* spp. and *Streptococcus lactis* were proved to detoxify patulin. Among them, *Bifidobacterium* VM12 displayed the best degradation effect; moreover, a HepG2 toxicity test demonstrated that *Bifidobacterium* VM12 greatly reduced the toxicity of patulin [195]. *L. acidophilus* has also shown a higher removal efficiency on PAT and OTA, and the factors such as the initial concentration of mycotoxin, pH, cell density and strain growth significantly influence the detoxification efficiency of *L. acidophilus* [196]. Some progress on trichothecenes detoxification was made by bacterial strains; the strain of commercialized *A. Eubacterium* (BBSH 797) could deactivate trichothecenes by reducing of the epoxide ring [197]. Treatment of DON with a strain of BBSH 797 produced a de-epoxy DON of DOM-1, whose IC_50_ value was 54-fold lower than that of DON, while the IC50 of de-epoxy NIV is 55-fold lower than that of NIV [198].

In addition, the bacteria strains of LS100 and SS3 that were isolated from chicken intestinal microbes have the ability to degrade 12 trichothecenes, and de-acylation and de-epoxidation are the main degradation pathways [199]. Furthermore, the hydroxylation and carbonylation of molecular structures markedly influence the toxicity of trichothecenes; for instance, the difference between NIV and DON is that NIV has C4-OH, which makes the toxicity of NIV 10 times higher than that of DON [200]. *Baccharis* spp. transformed T-2 toxin, in presence of NADPH, into HT-2 toxin with C3′-OH through a hydroxylation reaction, which mainly occurs in liver microsomes [201].

The yeast strains of *Pichia ohmeri*, *Saccharomyces cerevisiae*, *Rhodotorula* spp. and *Paecilomyces* spp. have shown the capacity to degrade mycotoxins. *S. cerevisiae* can degrade patulin from apple juice under oxygen-free conditions. *R. paludigenum* treatment completely degraded patulin in vitro at 30 °C and pH at 6.0 for 48 h, and the involved action mechanism was mainly attributed to enzymolysis [202]. Li et al. reported that patulin was degraded into desoxypatulinic acid by *R. mucilaginosa* JM19; in addition, the capacity of *R. mucilaginosa* JM19 to degrade patulin was strongly dependent on temperature, cell density and initial patulin concentration [203]. Tang et al. suggested the main action mechanism is that *R. mucilaginosa* secreted a kind of orotate phosphoribosyltransferase (EC: 2.4.2.10) in apple juice during the degradation process, which plays an important role in the degradation of patulin [204]. Xing et al. cloned a short-chain dehydrogenase/reductase (SDR) gene of CgSDR from the yeast strain of *Candida guilliermondii*, which was expressed in *Escherichia coli*, and the recombination expression of CgSDR conferred a strong tolerance and degradation capacity on patulin. The purified CgSDR transformed patulin into E-ascladiol with the help of the NADPH of a coenzyme. When 150 µg/mL of CgSDR was added into the apple juice contaminated with patulin, the degradation efficiency reached 80%; more importantly, the quality of the apple juice did not decrease during the treatment process [205].

Zhang et al. screened the optimum conditions of *Yarrowia lipolytica* degradation of OTA, which were 28 °C, pH 4.0, 108 spores/mL of yeast concentration and the degradation rate reaching 95.7% in vitro. In vivo, the same concentration of *Y. lipolytica* directly reduced 90% of the OTA content in the lesion part of infected table grapes [206]. The possible mechanism of OTA elimination by *Yarrowia lipolytica* Y-2 was analyzed by Zhang et al., who indicated that OTA degradation by *Y. lipolytica* was attributed to the action of intracellular enzymes, in which two kinds of carboxypeptidase proteins were expressed and reflected the hydrolysis activity of carboxypeptidase [207]. *Hanseniaspora uvarum* could also significantly and efficiently degrade OTA [208].

Porcine pancreatic lipase, a kind of hydrolases, can efficiently catalyze a variety of biological reactions. Tang et al. suggested that calcium carbonate immobilized porcine pancreatic lipase can be applied as an alternative biological strategy for patulin detoxification in apple juice; the optimal degradation condition is 40 °C, 18 h and calcium carbonate immobilized porcine pancreatic lipase of 0.03 g/mL [209]. Moreover, Liu et al. inferred the degradable product of patulin after the fermentation of porcine pancreatic lipase and patulin and indicated that the molecular weight is 159.0594, named C_7_H_11_O_4_ [210].

## 5. Conclusions

The diseases of fruits and vegetables caused by fungal infections are widely distributed, which has attracted great attention all over the world. Mycotoxins produced by fungi in fruits and vegetables mainly include ochratoxin A, patulin, Alternaria toxins and trichothecenes, and most of these have the potential to threaten human and animal health. As one of the most important dietary sources for human beings, it is essential to ensure the safety of fruits and vegetables. At present, in only a few varieties of fruits and vegetables have mycotoxins been systematically studied, especially for trichothecenes, which need further research. Accurate and rapid detection technology, risk assessment and degradation technology have become hot issues for the future. In addition to traditional methods that require large-scale instruments, biosensors and immunological techniques have also made a positive improvement and achieved good results. However, there are still some problems to be improved in the detection of mycotoxins. In fruits and vegetables, different mycotoxins often coexist, and it is necessary to develop a technique for the simultaneous detection of multiple mycotoxins. From the perspective of toxin control technology, the available physical and chemical strategies to remove mycotoxins from fruits and vegetables have some issues, such as safety issues, losses in nutrition and quality, environmental contamination and high cost, which limit their application at a large scale. Biodegradation, due to its features of high efficiency, specificity and non-pollution, is considered a powerful and potential alternative strategy and has aroused more and more concern. In addition, most research on detoxification is focused on the screening and isolation of microbial strains, and few studies contribute to the involved detoxification mechanism. Moreover, in future, we need to isolate and identify high-purity enzymes for the degradation of mycotoxins, analyze the protein structure and action mechanism and demonstrate the encoding gene of the degrading enzyme, to be expressed highly with the help of genetic engineering.

## Figures and Tables

**Table 1:** Contamination of ochratoxin A in fruits and vegetables.

	Foodstuffs	Detection Method	Country	Positives (%)	OTA (µg/Kg)	Ref.
Fruits and products	Peppers	HPLC-FD	Poland	37.5%	1.7–2.4	[26]
	Grapes	ELISA	Italy	30.4%	0.02–9.2	[30]
	Strawberries	HPLC-FD	China	-	-	[32]
	Apple juice	HPLC-FD	Saudi Arabia	29.41%	100–200	[33]
Dried fruits	Raisins	HPLC-FD	Poland	47%	1.1–34	[26]
	Figs	HPLC-FD	Turkey	48%	0.1–15.3	[34]
	Figs	ELISA	Iran	10.4	2.3–14.2	[35]
	Apricots	ELISA	Iran	6.7%	2.8	[35]
	Raisins	ELISA	Iran	44.7%	2.9–18.2	[35]
	Dates	ELISA	Iran	10%	1.4–3.6	[35]
	Figs	ELISA	Spain	54.3%	3.15–277	[36]
	Apricots	HPLC-UV–VIS	India	28.57%	194 ± 0.001	[37]
	Raisins	HPLC-FD	Pakistan	23.5%	5.60 ± 1.34	[38]
	Apricots	HPLC-FD	Pakistan	23.1%	3.10 ± 0.70	[38]
	Plums	HPLC-FD	Pakistan	25%	3.90 ± 0.95	[38]
	Figs	HPLC-FD	Pakistan	21.4%	2.10 ± 0.79	[38]
	Mulberries	HPLC-FD	Iran	45.5%	0.4–3.4	[39]
	Dates	HPLC-FD	Iran	22.3%	0.5–2.1	[39]
	Figs	HPLC-FD	Iran	45.5%	0.4–12.2	[39]
	Apricots	HPLC-FD	Iran	50%	0.75–5.5	[39]
Dried vegetables	Packed red peppers	HPLC-FD	Turkey	87.1%	0.6–1.0	[40]
	Unpacked red peppers	HPLC-FD	Turkey	100%	1.1–31.7	[40]
	Packed red peppers	HPLC-FD	Korea	48%	0.23–56.30	[41]
	Unpacked red peppers	HPLC-FD	Korea	4%	0.15–0.20	[41]

Note: “-” means no relevant report, and “0” means no detection; HPLC-FD: high-performance liquid chromatography with fluorescence detection; ELISA: enzyme-linked immunosorbent assay; HPLC-UV–VIS: high-performance liquid chromatography with variable wavelength absorbance detector.

**Table 2:** Contamination of patulin in fruits and vegetables.

	Foodstuffs	Detection Method	Country	Positives (%)	PAT (µg/Kg)	Ref.
**Fresh fruits**	Tomatoes	HPLC-UV	Belgium	10.8	-	[65]
	Sweet bell peppers	HPLC-UV	Belgium	11.4	-	[65]
	Onions	HPLC-UV	Belgium	-	-	[65]
	Apricots	HPLC-UV-VIS	Argentina	4.5	0.7	[29]
	Grapes	HPLC-UV-VIS	Argentina	10	28.3	[29]
	Pears	HPLC-UV-VIS	Argentina	10.7	54	[29]
	Peaches	HPLC-UV-VIS	Argentina	9.7	5	[29]
	Pineapples	HPLC-UV-VIS	Argentina	-	-	[29]
	Oranges	HPLC-UV-VIS	Argentina	50	0.1	[29]
	Seedless grapes	HPLC-UV	Pakistan	70	286.1	[66]
	Red globe grapes	HPLC-UV	Pakistan	75	921.1	[66]
	Flame grapes	HPLC-UV	Pakistan	66.7	190.1	[66]
	Pineapples	HPLC-UV	Pakistan	81.8	254.1	[66]
	Pears	HPLC-UV	Pakistan	66.7	232.1	[66]
	Tomatoes	HPLC-UV	Pakistan	80	410.2	[66]
**Dried fruits**	Figs	HPLC-UV	China	65	87.6	[68]
	Longans	HPLC-UV	China	90.5	68.4	[68]
	Apricots, dates, plums, peaches and bananas	HPLC-UV	China	8.3	7.4	[68]
	Apricots	HPLC-UV	Iraq	100	0.008–2.84	[69]
	Grapes	HPLC-UV	Iraq	100	0.0198–30.5	[69]
**Fruit products**	Pear juice	HPLC-UV	Tunisia	47.61%	62.5	[64]
	Pear jams	HPLC-UV	Tunisia	43.75%	123.7	[64]
	Fruit juice	HPLC-UV	Pakistan	58.3	110.3	[66]
	Smoothie of tomatoes, mint and carrots	HPLC-UV	Pakistan	44.4	50.7	[66]
	Smoothie of pineapple and watermelon	HPLC-UV	Pakistan	42.9	60.6	[66]
	Smoothie of oranges, carrots and mint	HPLC-UV	Pakistan	50	110.4	[66]
	Smoothie of banana, mangoes and strawberry	HPLC-UV	Pakistan	50	20.3	[66]
	Hawthorn products	HPLC-UV	China	10	5.1	[68]
	Fruit juice	HPLC-UV	China	15	5.4	[68]
	Fruit jams	HPLC-UV	China	10	5.0	[68]

Note: “-” means no relevant report, and “0” means no detection; HPLC-UV: high-performance liquid chromatography with ultraviolet detector.

**Table 3:** Contamination of Alternaria toxins in fruits and vegetables.

	Foodstuffs	Detection Method	Country	AOH		AME		TeA		Ref.
				Positives (%)	AOH (µg/Kg)	Positives (%)	AME (µg/Kg)	Positives (%)	TeA (µg/Kg)	
**Fresh fruits**	Apples	LC–MS/MS	China	27.88	6.71–8517	16.35	4.97–2623	66.35	36–145276	[9]
	Apples	LC–MS/MS	Netherlands	-	0	-	0	-	0	[88]
	Tomatoes	LC–MS/MS	Netherlands	-	0	-	0	-	0	[88]
	Grapes	UPLC–MS/MS	China	26.8%	0.09–7.15	3.6%	0.11–0.15	28.6%	0.25–46.97	[91]
**Dried fruits**	Raisins	UPLC–MS/MS	China	5.3	3.5~15.6	19.3	0.3~13.5	35.1	6.9~594.4	[91]
	Dates	UPLC–MS/MS	China	-	-	-	-	34.0	9.6~4411.4	[91]
	Apricots	UPLC–MS/MS	China	-	-	5.4	0.5~2.1	37.5	10.4~1231.8	[91]
	Figs	LC–MS/MS	Netherlands	0	-	0	-	100	25–2345	[88]
	Wolfberries	UPLC–MS/MS	China	3.7	5.9~27.4	7.4	0.2~15.0	64.8	23.8~5665.3	[91]
**Fruits products**	Apple juice	LC–MS/MS	China	2.44	3.70	4.88	<LOQ	9.76	<LOQ	[9]
	Apple jams	LC–MS/MS	China	23.53	<LOQ-4.4	-	-	-	-	[9]
	Apple vinegar	LC–MS/MS	China	2.94	<LOQ	-	-	2.94	14.5	[9]
	Juice	EIA	Germany	56.5	0.65–16	43.5	0.14–4.9	52.2	21–250	[92]
	Red wine	LC-UV and LC–MS/MS	Canada	83.3	0.03–19.4	83.3	0.01–0.23	-	-	[93]
	Tomato sauce	HPLC–MS/MS	Belgium	86	<LOQ-42	78	<LOQ-3.8	84.3	7.7–330.6	[94]
	Tomato concentrate	HPLC–MS/MS	Belgium	85	<LOQ-31	67	<LOQ-6.1	100	<LOQ-174	[94]
	Tomato juice	HPLC–MS/MS	Belgium	71	<LOQ-7.0	54	<LOQ-3.3	100	3.7–333.1	[94]
	Trockenbeerenauslese	LC–MS/MS	Germany	66.7	1.2–4.9	66.7	0.1–0.3	-	-	[95]
	Vegetable products	LC–MS/MS	Germany	50	2.6–25	60	0.1–5			[95]

Note: “-” means no relevant report, and “0” means not detected; EIA: implementation in enzyme immunoassay.

**Table 4:** Sequence of mycotoxin aptamers.

Mycotoxin	Sequence	Dissociation Constant (nmol/L)	Reference
OTA	GATCGGGTGTGGGTGGCGTAAAGGGAGCATCGGACA	200	[139]
PAT	GGCCCGCCAACCCGCATCATCTACACTGAT ATTTTACCT T	21.83 ± 5.022	[138]
ZEN	CGTGCTACCGTGAAATACCAGCTTATTCAATTCTACCAGCTTTG AGGCTCGATCCAGCTTATTCAATTATACCAGCTTATTCAATTATACCAGCACAATCGTAATCAGTTAG	15.2 ± 3.4	[138]
FB1	ATACCAGCTTATTCAATTAATCGCATTACCTTATACCAGCTTATTCAATTACGTCTGCACATACCAGCTTATTCAATTAGATAGTAAGTGCAATCT	100 ± 30	[139]
T-2	GTATATCAAGCATCGCGTGTTTACACATGCGAGAGGTGAA	20.8 ± 3.1	[140]
